# Reference ranges of computed tomography-derived strains in four cardiac chambers

**DOI:** 10.1371/journal.pone.0303986

**Published:** 2024-06-06

**Authors:** Yura Ahn, Hyun Jung Koo, Seung Ah Lee, DaSol Jung, Joon-Won Kang, Dong Hyun Yang

**Affiliations:** 1 Department of Radiology and Research Institute of Radiology, Republic of Korea; 2 Division of Cardiology, Asan Medical Center, University of Ulsan College of Medicine, Seoul, Republic of Korea; All India Institute of Medical Sciences, INDIA

## Abstract

Research on cardiovascular diseases using CT-derived strain is gaining momentum, yet there is a paucity of information regarding reference standard values beyond echocardiography, particularly in cardiac chambers other than the left ventricle (LV). We aimed to compile CT-derived strain values from the four cardiac chambers in healthy adults and assess the impact of age and sex on myocardial strains. This study included 101 (mean age: 55.2 ± 9.0 years, 55.4% men) consecutive healthy individuals who underwent multiphase cardiac CT. CT-derived cardiac strains, including LV global and segmental longitudinal, circumferential, and transverse strains, left atrial (LA), right atrial (RA), and right ventricle (RV) strains were measured by the commercially available software. Strain values were classified and compared by their age and sex. The normal range of CT-derived LV global longitudinal strain (GLS), global circumferential strain (GCS), and global radial strain (GRS) were −20.2 ± 2.7%, −27.9 ± 4.1%, and 49.4 ± 12.1%, respectively. For LA, reservoir strain, pump strain, and conduit strain were 28.6 ± 8.5%, 13.2 ± 6.4%, and 15.5 ± 8.6%, respectively. The GLS of RA and RV were 27.9 ± 10.9% and −22.0 ± 5.7%, respectively. The absolute values of GLS of RA and RV of women were higher than that in men (32.4 ± 11.4 vs. 24.3 ± 9.1 and −25.2 ± 4.7 vs. −19.4 ± 5.0, respectively; p<0.001, both). Measurement of CT-derived strain in four cardiac chambers is feasible. The reference ranges of CT strains in four cardiac chambers can be used for future studies of various cardiac diseases using the cardiac strains.

## Introduction

Although echocardiography is the most used modality for cardiac function assessment, but its representativeness has been questioned due to its low sensitivity for subclinical myocardial dysfunction and observer variability [1]. Meanwhile, myocardial strain, a dimensionless measurement demonstrating the deformation of the myocardium during the cardiac cycle, has been highlighted across the various cardiac diseases from ischemic [2, 3] to nonischemic cardiomyopathy [4, 5] and valvular heart disease [6, 7] as an indicator of disease status and its prognostic value.

Currently, echocardiography and cardiac magnetic resonance imaging (CMR) were established modalities for assessing cardiac strain. Recent advances in echocardiography enable deformity measurement in any direction with a 3-dimensional speckle tracking method despite low spatial resolution and dependency on the vendor and observer. CMR can accurately measure, particularly with the tagging method, but has limited accessibility, high cost, and demands an extended processing time [8]. On the other hand, since strain measurement using cardiac computed tomography (CT) via a feature tracking method was reported in 2014 [9], a series of studies have demonstrated the feasibility of CT-derived strain in valvular heart disease [10–12], ischemic cardiomyopathy [13], and congestive heart failure [9].

CT-derived strain has advantages in that concomitant evaluation of coronary artery disease with CT coronary angiography is enabled, and other cardiac chambers that are hard to evaluate with echocardiography are visualized by high spatial resolution and more easily accessible than CMR. With increasing utilization of CT coronary angiography, the use of CT-derived strain is expected to grow. Still, we have little knowledge of the normal value of CT-derived cardiac strain. Only a few studies measured the strain with CT in a normal population and reported results for left ventricular (LV) myocardium alone [14, 15]. However, the prognostic implications of strains in the other cardiac chambers, such as the right ventricle (RV) [16] or left atrium (LA) [17–19] has been actively investigated, assessment of feasibility and the normal value of CT-derived strains in these chambers would be timely and meaningful. Therefore, in this study, we investigated the reference ranges of CT-derived strain in all four cardiac chambers and explored the influence of age and sex on the normal cardiac stains.

## Materials and methods

### Study population

The institutional review board (IRB) of [BLINDED] approved this retrospective study and waived the requirement for informed patient consent (approval number: 2022–0903). The research data were accessed for research purposes after obtaining IRB approval in July 2022. From January 2014 to August 2014, consecutive asymptomatic individuals who visited the health screening center of a tertiary hospital, voluntarily underwent multiphase cardiac CT, and identified no significant coronary artery stenosis were retrospectively included in this study. All study subjects were screened for cardiovascular symptoms, signs, and risk factors, and received transthoracic echocardiography (TTE) using commercially available echocardiographic systems (iE33 and Epiq 7 or Vivid 7 and E9 [GE Healthcare]). Details of the measurement of echocardiographic strain values are described in S1 Text. We excluded patients with a history of diabetes mellitus (n = 6) and abnormal results on echocardiography (n = 1), and finally included 101 healthy subjects. In our study, 12 subjects who had minimal (n = 10) or mild (n = 2) coronary stenosis on cardiac CT were not excluded based on their normal echocardiography results. No patient was diagnosed with hypertension at the time of CT acquisition.

### Cardiac CT acquisition and measurement of CT-derived cardiac strain

**Fig 1:**
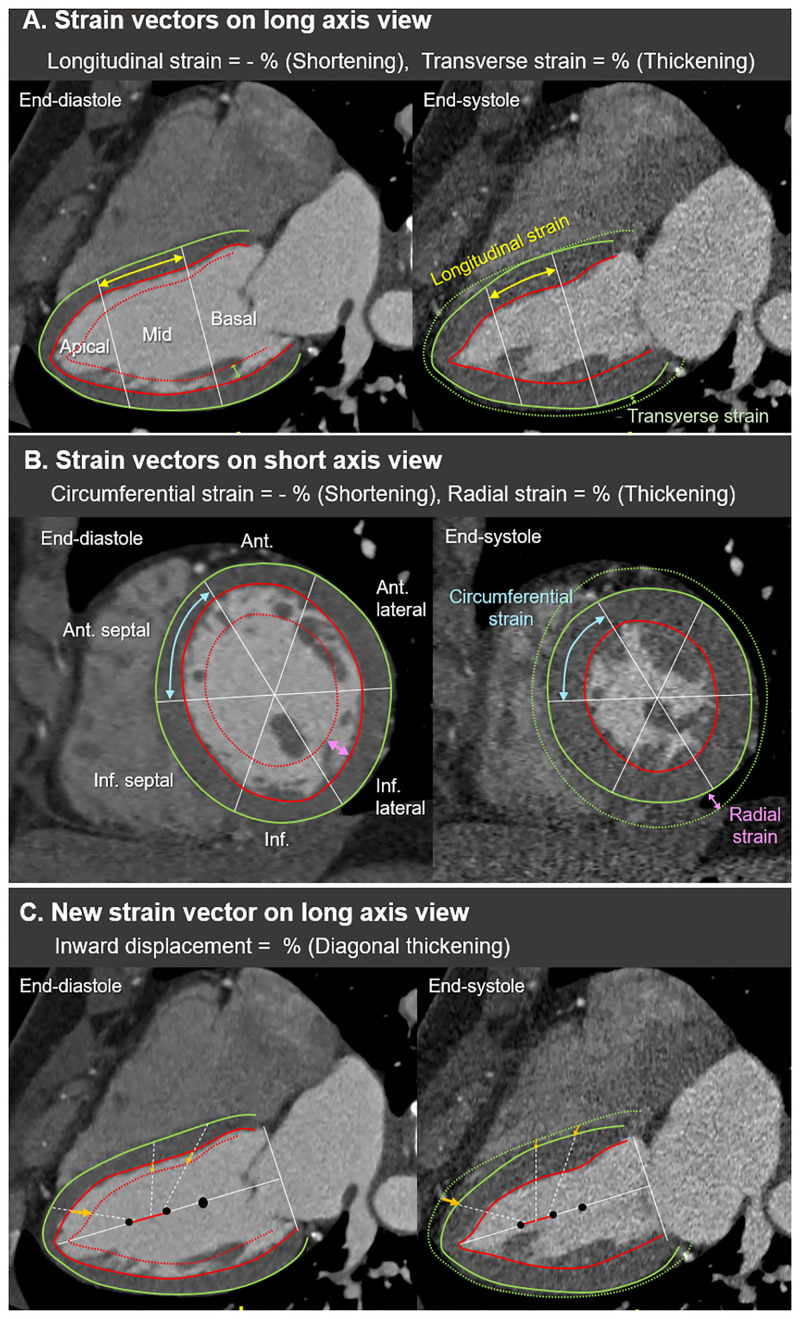


**Fig 2:**
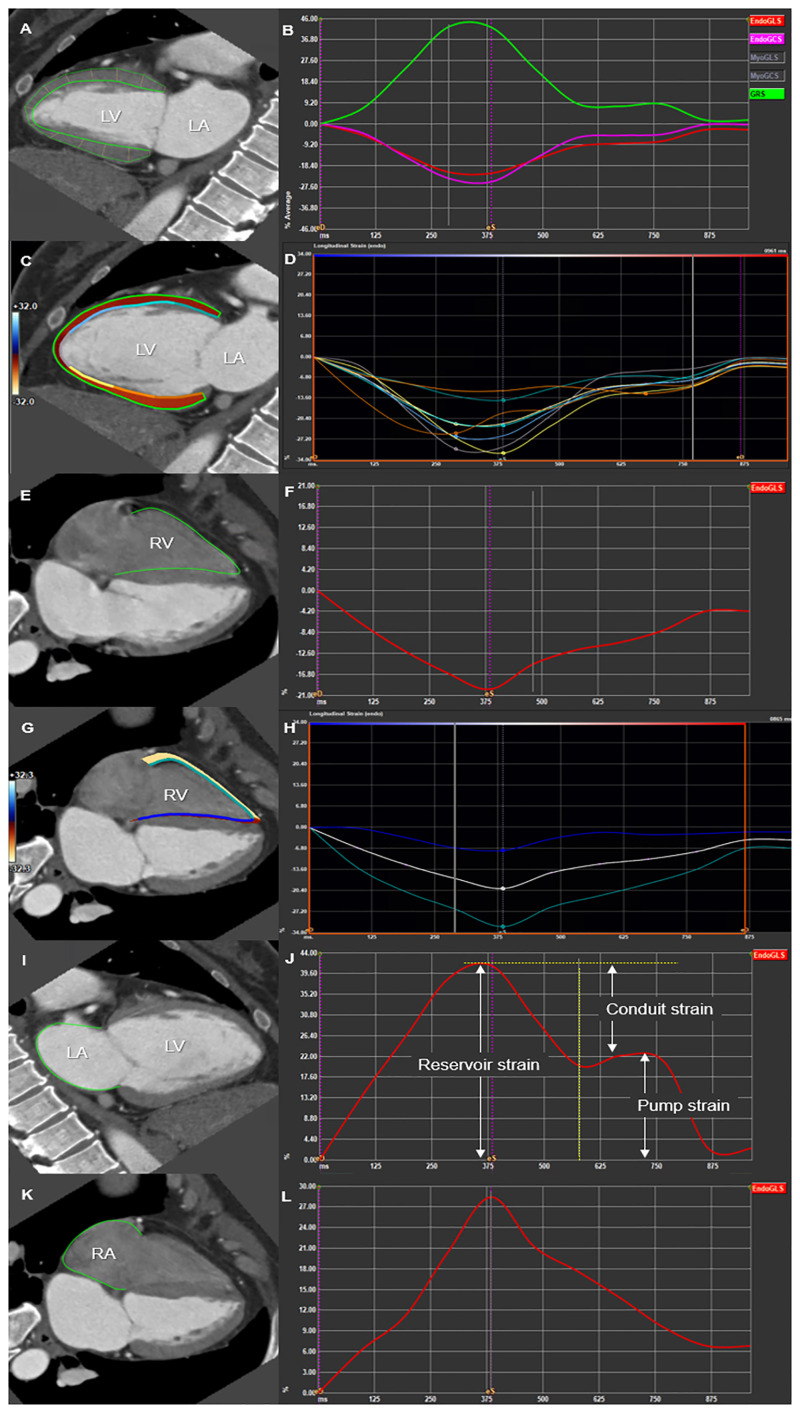


Electrocardiography (ECG)-gated cardiac CT was performed using a second-generation dual-source CT scanner (Somatom Definition Flash, Siemens, Erlangen, Germany). Details of the CT protocols are provided in S2 Text. The commercially available software (Medis suite, version 4.0, Leiden, The Netherlands) was used to measure the CT-derived cardiac strain. The graphical summary and detailed description of measurement for the strain of each chamber are demonstrated in Figs 1 and 2, S3 Text, and S1 and S2 Figs.

### Statistical analysis

Characteristics of the subjects according to the age and sex were analyzed using the independent t-test or Mann–Whitney *U* test for continuous variables and the chi-square test or Fisher exact test for categorical variables. The normality of the continuous variable was checked by the Shapiro-Wilk test. Correlations between CT-derived strain parameters and the age were evaluated using the Pearson correlation test. The strain parameters across the segments were compared using a one-way analysis of variance. If a significant difference between the regions was identified, a specific region was analyzed using a post hoc test with the Scheffe Test. Interobserver agreements were evaluated using a Bland-Altman method. All statistical analyses were performed using the MedCalc statistical software (version 20.111) and R (version 4.2.0), with p-values of <0.05 defined as statistically significant.

## Results

### Patient characteristics

A total of 101 healthy subjects were finally included (mean age, 55.2±9.0 years, 56 men [55.4%]). Significant differences were observed in weight, height, body surface area, and body mass index between men and women (all, p<0.05). In echocardiography, both LV internal diameter at end diastole and systole, LV end-systolic volume, and LV end-diastolic volume were significantly lower in women than in men (all, p<0.05) (Table 1).

**Table 1:** Patient characteristics.

Parameters	Total (n = 101)	Men (n = 56)	Women (n = 45)	*P*
Age, years	55.2 ± 9.0	54.9 ± 8.1	55.5 ± 10.1	0.738
Weight, kg^a^	64.6 (57.4–72.7)	70.7 (64.4–76.7)	54.6 (51.6–63.2)	< .001
Height, cm^a^	164.7 (159.5–172.6)	169.6 (165.4–174.3)	159.2 (155.4–162.5)	< .001
Body surface area, m^2^^a^	1.7 ± 0.2	1.8 ± 0.2	1.6 ± 0.1	< .001
Body mass index, kg/m^2^^a^	23.7 (21.9–25.8)	25.1 (22.3–26.1)	22.3 (20.9–24.5)	0.010
Systolic blood pressure, mmHg	125.4 ± 15.7	125.2 ± 16.3	125.6 ± 15.2	0.899
Diastolic blood pressure, mmHg	78.3 ± 10.5	79.4 ± 10.9	76.9 ± 10.0	0.228
LDL cholesterol, mg/dL	125.5 ± 35.1	128.6 ± 35.6	120.6 ± 34.4	0.311
HbA1c, %	5.5 (5.3–5.8)	5.6 (5.3–5.8)	5.5 (5.4–5.6)	0.454
**Echocardiography**				
LVIDs, mm	29.3 ± 4.3	30.6 ± 4.6	27.5 ± 3.2	0.001
LVIDd, mm	47.3 ± 4.7	48.7 ± 4.2	45.3 ± 4.7	0.001
LV mass index, g/m^2^^a^	79.8 (68.6–98.4)	84.4 (70.1–99.2)	77.5 (65.2–88.7)	0.234
LV ESV, ml^a^	32.0 (26.0–37.0)	35.0 (29.0–40.0)	29.0 (22.0–32.0)	0.006
LV EDV, ml^a^	85.0 (72.0–97.0)	90.0 (77.0–101.0)	77.0 (68.0–90.0)	0.018
LVEF, %	62.4 ± 4.1	61.7 ± 3.6	63.3 ± 4.7	0.075

Unless otherwise indicated, data are mean ± standard deviation.

^a^Data in parentheses are ranges

^b^Data in parentheses are percentages

EDV = end-diastolic volume, ESV = end-systolic volume, LDL = low-density lipoprotein, LV = left ventricular, LVEF = left ventricular ejection fraction, LVIDd = left ventricular internal diameter at end diastole, LVIDs = left ventricular internal diameter at end systole

### Global strain in four cardiac chambers

**Fig 3:**
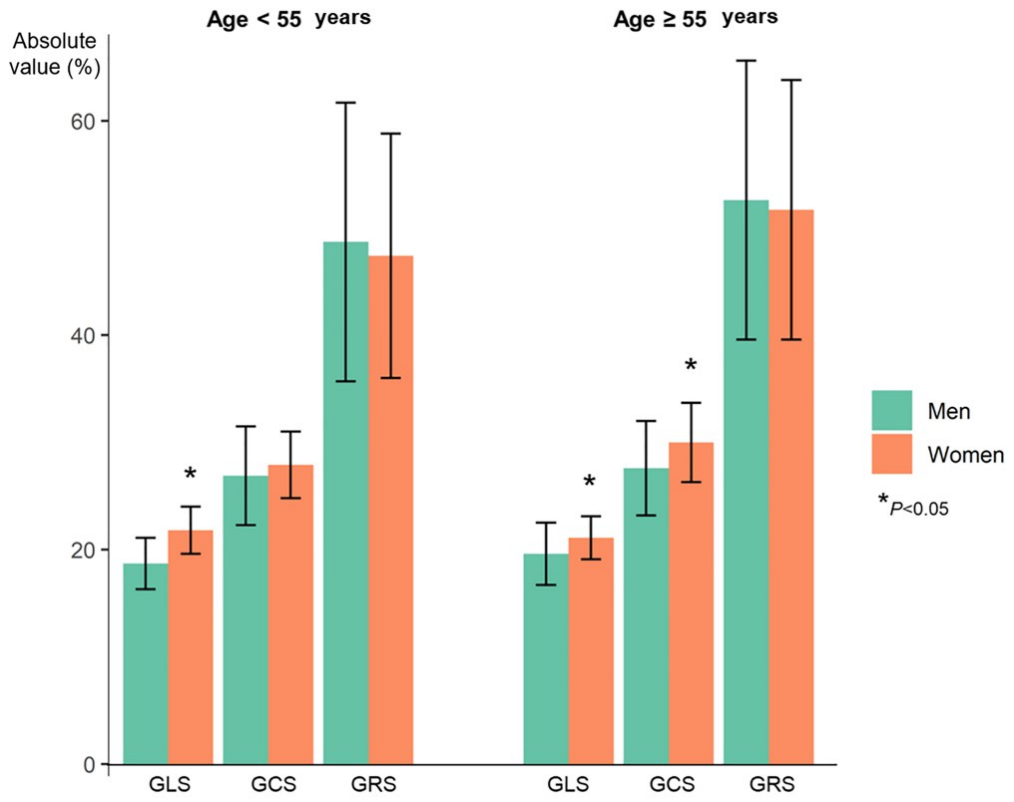


The mean values of LV GLS, GCS, and GRS were −20.2±2.7%, −27.9±4.1%, and 49.4±12.1%, respectively. The median of absolute value of LV GLS and GCS were significantly lower in women than those in men (median, −18.7% vs. −21.6%, p<0.001 and −26.9% vs. −28.7%, p = 0.005, respectively), whereas LV GRS showed no significant difference (Table 2). LV strain values are presented by the age and sex (Age <55 vs. ≥55 years [Table 3 and Fig 3], and 20–39 vs. 40–59 vs. ≥60 years [S3 Fig]). LV GLS and GCS showed weak negative correlations with the age in men and women (r = −0.27, p = 0.045; and r = −0.30, p = 0.042, respectively) (Table 3).

**Table 2:** CT measurements and strain parameters.

	Total Mean± SD	Total Median (IQR)	Men (n = 56) Mean ± SD	Women (n = 45) Mean ± SD	Men (n = 56) Median (IQR)	Women (n = 45) Median (IQR)	*P* ^a^
**Left ventricle**							
Global longitudinal strain, %	−20.2 ± 2.7	−20.0 (−22.0–−18.4)	−19.0 ± 2.6	−21.6 ± 2.0	−18.7 (−19.9–−17.6)	−21.6 (−22.7–−20.4)	< .001^b^
Global circumferential strain, %	−27.9 ± 4.1	−27.9 (−30.1–−25.2)	−26.9 ± 4.4	−29.0 ± 3.5	−26.9 (−28.8–−24.1)	−28.7 (−31.2–−26.2)	0.005^b^
Global radial strain, %	49.4 ± 12.1	48.1 (40.6–57.8)	49.8 ± 12.8	49.0 ± 11.4	48.2 (40.5–58.9)	47.3 (40.6–57.1)	0.735^c^
EF, %	56.2 ± 5.4	55.7 (52.7–59.6)	54.3 ± 5.6	58.5 ± 4.1	54.0 (50.7–56.0)	58.1 (55.7–61.2)	< .001^b^
**Right ventricle**							
Global longitudinal strain, %	−22.0 ± 5.7	−21.8 (−26.1–−18.2)	−19.4 ± 5.0	−25.2 ± 4.7	−19.2 (−23.3–−16.2)	−25.8 (−27.9–−21.8)	< .001^c^
RV free wall strain, %	−26.9 ± 6.4	−27.7 (−30.6–−22.8)	−23.9 ± 5.6	−30.7 ± 5.3	−24.1 (−29.2–−19.4)	−29.8 (−33.5–−27.9)	< .001^c^
RV septal strain, %	−16.2 ± 6.5	−15.6 (−21.2–−11.4)	−13.6 ± 5.5	−19.3 ± 6.5	−14.1 (−17.2–−9.6)	−19.1 (−23.7–−15.2)	< .001^c^
FAC, %	36.3 ± 7.7	36.4 (32.7–40.8)	33.1 ± 7.2	40.3 ± 6.2	34.3 (28.9–36.2)	40.1 (37.2–42.7)	< .001^b^
**Left atrium**							
LA reservoir strain, %	28.6 ± 8.5	26.7 (23.2–33.0)	27.7 ± 8.6	29.8 ± 8.3	25.9 (21.1–32.6)	27.5 (24.3–33.6)	0.130^b^
LA pump strain, %	13.2 ± 6.4	14.2 (9.5–17.4)	13.9 ± 5.8	12.3 ± 7.0	14.3 (10.1–17.2)	13.1 (7.2–17.8)	0.236^c^
LA conduit strain, %	15.5 ± 8.6	13.8 (10.2–18.1)	13.8 ± 8.0	17.5 ± 8.8	12.9 (9.3–16.0)	16.6 (12.1–20.9)	0.010^b^
LA volume, mL	72.6 ± 19.1	72.2 (60.2–83.2)	71.8 ± 17.3	73.5 ± 21.2	72.1 (60.9–83.5)	72.2 (59.4–83.2)	0.864^b^
FAC, %	36.1 ± 6.6	35.6 (31.5–39.8)	35.1 ± 6.9	37.2 ± 6.1	35.0 (30.9–39.9)	37.0 (32.6–39.7)	0.105^c^
EF, %	48.9 ± 8.0	48.8 (43.2–54.1)	47.4 ± 8.5	50.8 ± 7.1	46.4 (42.0–53.2)	51.7 (45.9–54.4)	0.037^c^
**Right atrium**							
Global longitudinal strain, %	27.9 ± 10.9	26.8 (19.9–36.2)	24.3 ± 9.1	32.4 ± 11.4	23.5 (18.0–28.1)	31.9 (25.3–41.7)	< .001^c^
RA volume, mL	60.2 ± 19.3	60.5 (48.1–72.0)	63.6 ± 21.1	55.9 ± 15.9	63.4 (51.4–74.0)	59.8 (45.7–66.4)	0.044^c^
FAC, %	33.6 ± 8.5	33.4 (26.7–40.2)	30.5 ± 7.8	37.5 ± 7.8	30.5 (24.9–34.7)	38.0 (33.2–42.9)	< .001^c^
EF, %	40.4 ± 10.0	39.7 (33.7–47.3)	36.5 ± 9.6	45.1 ± 8.5	37.3 (28.6–42.3)	45.4 (38.5–50.5)	< .001^c^

^a^For differences between sex

^b^Results of Mann–Whitney U test

^c^Results of independent t-test,

EF = ejection fraction, FAC = fraction area change, IQR = interquartile range, LA = left atrium, LV = left ventricle, RA = right atrium, RV = right ventricle, SD = standard deviation

**Table 3:** CT measurement and strain parameters according to the age and sex.

	Age < 55 (n = 50)			Age ≥ 55 (n = 51)			Men		Women	
	Men (n = 28)	Women (n = 22)	*P*	Men (n = 28)	Women (n = 23)	*P*	r	*P*	r	*P*
**Left ventricle**										
Global longitudinal strain, %	−18.5 ± 2.3	−21.9 ± 2.2	< .001	−19.4 ± 2.9	−21.4 ± 1.8	0.005	−0.27	0.045	0.07	0.645
Global circumferential strain, %	−26.5 ± 4.5	−28.0 ± 3.2	0.194	−26.9 (−29.4–−24.3)	−30.0 (−31.9–−28.2)	0.01	−0.21	0.124	−0.30	0.042
Global radial strain, %	47.7 ± 12.9	47.2 ± 11.7	0.873	51.8 ± 12.5	50.7 ± 11.1	0.729	0.24	0.069	0.24	0.113
EF, %	53.6 ± 6.1	57.5 ± 4.0	0.012	55.0 ± 5.2	59.4 ± 4.1	0.002	0.25	0.066	0.27	0.076
**Right ventricle**										
Global longitudinal strain, %	−18.2 ± 4.5	−25.1 ± 4.9	< .001	−20.5 ± 5.3	−25.4 ± 4.7	0.001	−0.31	0.020	−0.12	0.421
RV free wall strain, %	−23.7 ± 5.7	−32.4 ± 5.0	< .001	−24.1 ± 5.6	−29.1 ± 5.2	0.002	−0.11	0.432	0.21	0.169
RV septal strain, %	−12.2 ± 4.6	−17.6 ± 6.9	0.002	−15.1 ± 5.9	−21.0 ± 5.7	0.001	−0.31	0.020	−0.32	0.033
FAC, %	32.5 ± 5.0	40.6 ± 5.0	< .001	33.6 ± 9.0	40.1 ± 7.3	0.008	0.18	0.196	0.10	0.523
**Left atrium**										
LA reservoir strain, %	25.4 (20.5–32.4)	31.8 (26.6–40.1)	0.019	27.9 ± 7.3	26.8 ± 6.4	0.561	−0.06	0.680	−0.43	0.004
LA pump strain, %	13.5 ± 6.9	13.6 ± 7.4	0.963	14.2 ± 4.5	11.1 ± 6.5	0.053	−0.06	0.663	−0.24	0.118
LA conduit strain, %	12.9 (9.3–16.3)	17.7 (14.7–23.0)	0.009	12.2 (9.3–16.0)	14.5 (10.5–18.0)	0.323	−0.02	0.900	−0.22	0.156
LA volume, mL	71.6 ± 20.7	75.8 ± 18.3	0.465	72.5 (60.8–79.7)	70.7 (57.1–77.5)	0.369	−0.02	0.908	0.06	0.682
FAC, %	34.3 ± 7.4	39.6 ± 6.1	0.009	35.9 ± 6.3	34.9 ± 5.3	0.579	0.00	0.973	−0.42	0.004
EF, %	46.4 ± 9.3	53.5 ± 7.1	0.005	48.5 ± 7.6	48.2 ± 6.1	0.88	0.05	0.738	−0.39	0.009
**Right atrium**										
Global longitudinal strain, %	23.5 ± 9.7	30.3 ± 9.2	0.017	25.1 ± 8.5	34.5 ± 13.1	0.005	0.02	0.857	0.32	0.031
RA volume, mL	62.8 ± 21.9	56.7 ± 12.2	0.223	64.5 ± 20.6	55.1 ± 19.0	0.099	0.10	0.477	0.13	0.378
FAC, %	29.9 ± 8.0	35.9 ± 6.1	0.005	31.1 ± 7.7	38.9 ± 8.9	0.001	−0.01	0.963	0.33	0.025
EF, %	36.2 ± 9.7	42.5 ± 6.6	0.012	36.9 ± 9.6	47.6 ± 9.5	0.001	−0.02	0.903	0.39	0.008

Data in parenthesis are interquartile range

EF = ejection fraction, FAC = fraction area change, IQR = interquartile range, LA = left atrium, LV = left ventricle, RA = right atrium, RV = right ventricle, SD = standard deviation

In RV, the mean values of GLS, free wall strain, and septal strain were −22.0%, −26.9%, and −16.2%, respectively. All derived absolute values of RV strain parameters were significantly lower in men than those in women (mean, RV GLS, −19.4% vs. −25.2%; RV free wall strain, −23.9% vs. −30.7%; and RV septal strain, −13.6% vs. −19.3%, respectively, all p<0.001). RV GLS showed a weak negative correlation with the age in men (r = −0.31, p = 0.020) and RV septal strain also presented a weak negative correlation with the age in both men and women (r = −0.31, p = 0.020, and r = −0.32, p = 0.033, respectively).

The mean values of LA reservoir strain, pump strain, and conduit strain were 28.6%, 13.2%, and 15.5%, respectively. LA conduit strain showed a significant difference according to the sex (median, 12.9% in men vs. 16.6% in women, p = 0.010). LA reservoir strain and FAC had negative correlations with the age in women (r = −0.43, p = 0.004, and r = −0.42, p = 0.004, respectively).

**Fig 4:**
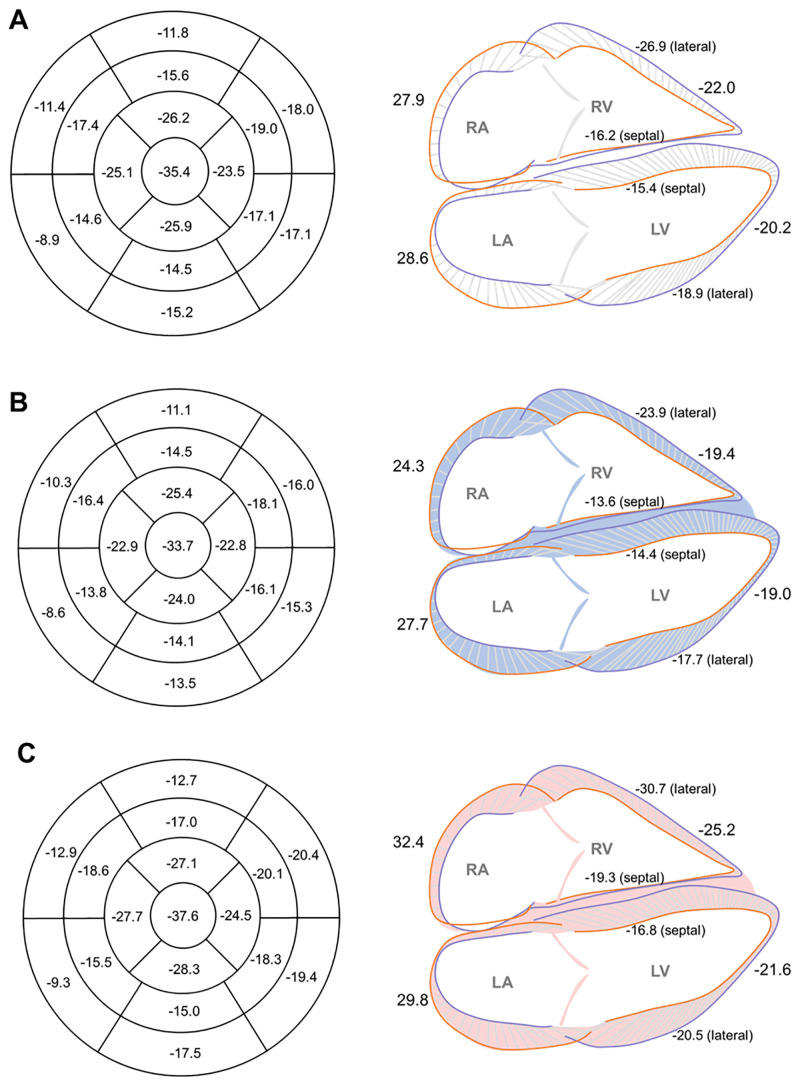


In RA, the mean value of RA GLS was 27.9%, and was significantly lower in men than those in women (mean, 24.3% vs. 32.4%, p< 0.001, and median, 23.5% vs. 31.9%). Moreover, a weak positive correlation was observed between RA GLS and the age in women (r = 0.32, p = 0.031, respectively). Similarly, RA FAC also showed a weak correlation with the age in women (r = 0.33, p = 0.025). The GLS values of all four cardiac chambers by each sex are summarized in Fig 4.

### Regional strain and inward displacement in LV

**Fig 5:**
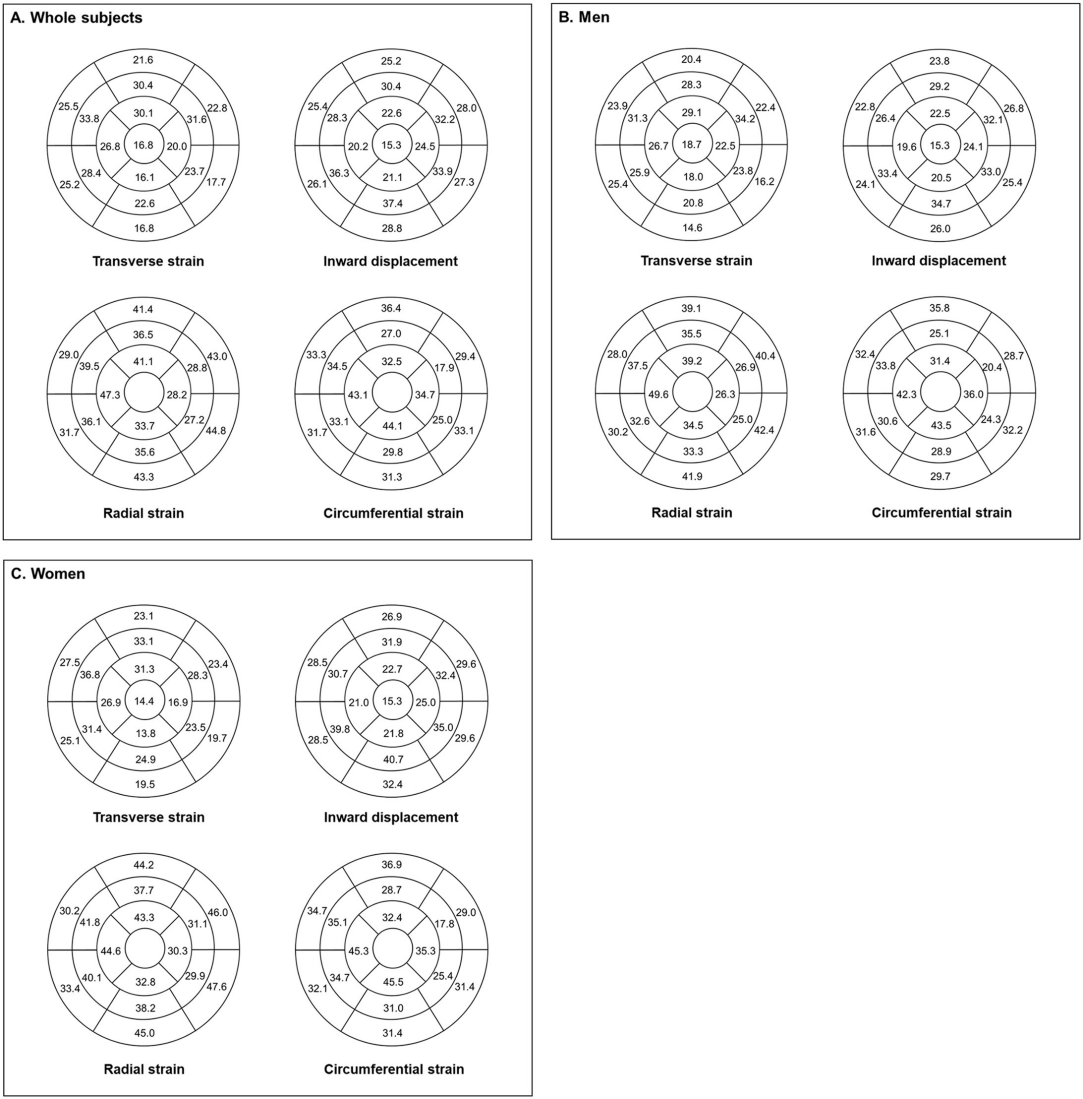


For LV segmental longitudinal strain, the values gradually increased from basal to apical segments. The values were largest in apical segments, with the maximum absolute value noted in LV apex (−35.4%) (Table 4 and Fig 4). Regional strains also had differences between LV lateral and septal walls. LV lateral walls consistently showed significantly higher absolute strain values than septal walls (Fig 4, all p< 0.001). On the other hand, LV transverse stain was largest in the mid segments (mean, 28.4%) with the largest value of 33.8% in the mid-anteroseptal segment (Fig 5). Similarly, LV inward displacement showed the largest values in mid segments (mean, 33.1%), with the maximum value in the mid-inferoseptal segment (36.3%) (Fig 5).

**Table 4:** Segmental strain values in different left ventricular regions.

	Basal segments	Middle segments	Apical segments	*P*
Peak longitudinal strain, %	−13.8 ± 3.0^a^^,^^b^	−16.4 ± 3.4^b^^,^^c^	−25.2 ± 4.1^a^^,^^c^	< .001
Transverse strain, %	21.6 ± 7.2^a^	28.4 ± 7.9^b^^,^^c^	23.2 ± 7.7^a^	< .001
Inward displacement, %	26.8 ± 4.0^a^^,^^b^	33.1 ± 4.7^b^^,^^c^	22.1 ± 5.1^a^^,^^c^	< .001

^a^ p < 0.05 compared with middle segments

^b^ p < 0.05 compared with apical segments

^c^ p < 0.05 compared with basal segment

### Interobserver reproducibility of strain analysis

**Fig 6:**
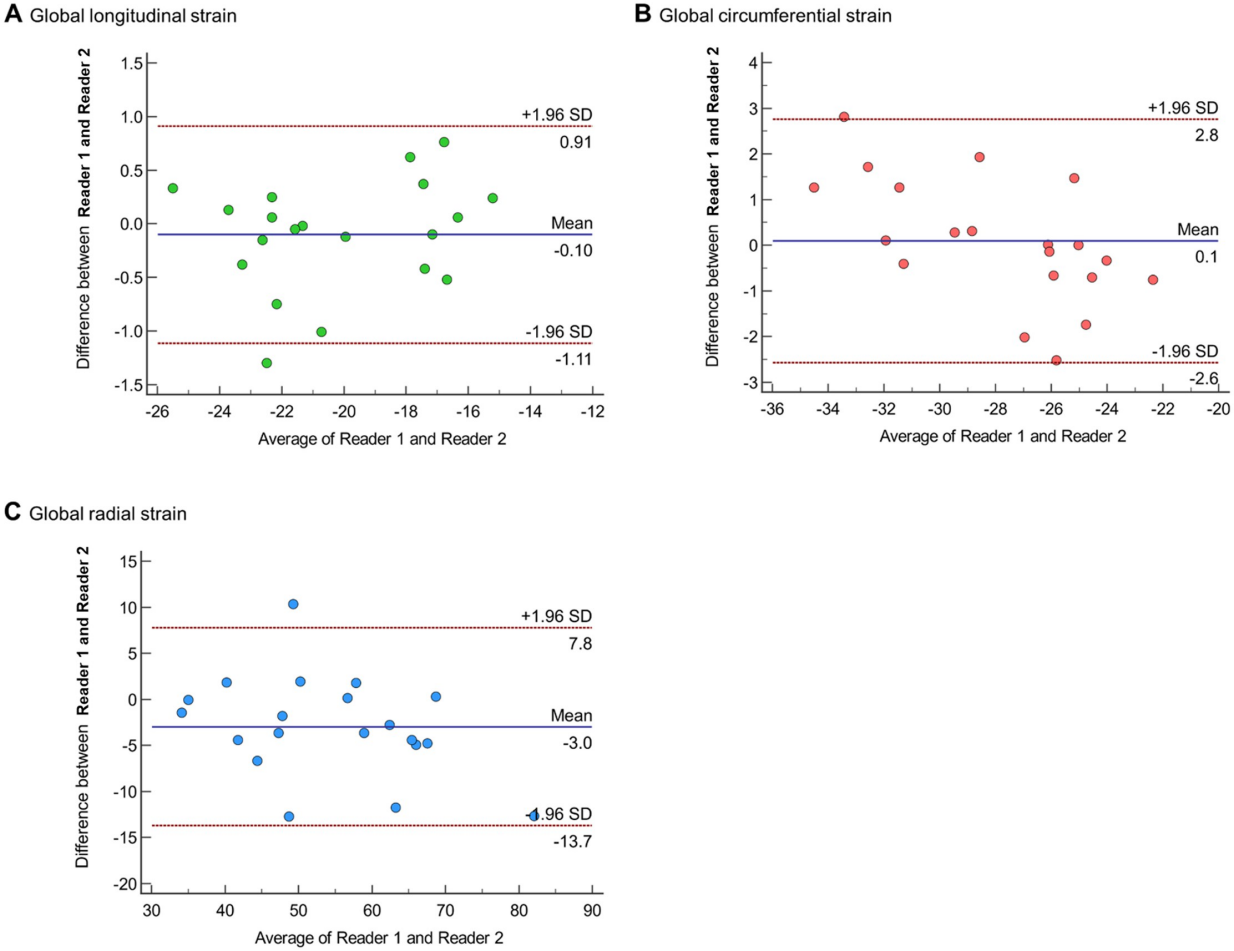


Fig 6 and S1 Table show the result of interobserver reproducibility between two readers for four cardiac chamber strain analyses. There was no demonstrable tendency in measured parameters from each observer, and the difference between the two observers was low, with the mean differences ranging from 0.0 to 2.8 and the absolute relative difference ranging from 0.0% to 7.7% overall, except in LV GRS and RV septal strain. The mean and absolute relative differences were largest for LV GRS, which were −14.3 (95% limits of agreement [LOA], −39.5–10.9) and −38.0%, respectively. The mean difference for the RV septal strain was slight to −0.1 (95% LOA, −5.9–5.8), but the absolute relative difference was 12.7%.

### Comparison with echocardiography-derived strain

Echocardiography 2D strain values were available in 34 patients (S4 Fig). CT strain slightly underestimated LV GLS (mean difference of −0.8, 95% LOA of [−5.0, 3.3] and showed moderate correlation with echocardiography-derived strain (r = 0.525, p = 0.001). Other parameters (LA reservoir, pump, conduit strains, LA FAC, RV GLS and RV FAC) showed mean difference ranged from −4.6 to 3.2, 95% LOA ranged from [−21.2, 11.9]to [−7.4, 13.8] and moderate correlation (r from 0.405 to 0.589).

## Discussion

This study demonstrated the reference ranges of CT-derived strains in four cardiac chambers using a feature tracking method. The reference range for CT strain of LV GLS, GCS, and GRS was −20.2±2.7%, −27.9±4.1%, and 49.4±12.1%, respectively. The absolute values of both LV GLS and GCS were higher in women and increased with age in men and women, respectively. The absolute values of GLS of RV and RA were also higher in women. Interobserver reproducibility was acceptable in all four chambers except the LV GRS and RV septal wall.

The measurement of cardiac strain using CT is being investigated in various diseases with increasing utilization of cardiac CT and its high spatial resolution and improving temporal resolution. As cardiac CT used in planning myomectomy for hypertrophic cardiomyopathy [20, 21] and transcatheter aortic valve implantation [22, 23], CT stain allows for the direct comparison and recognition of changes or improvements in strain through follow-up scans. Additionally, it also enables that the concurrent evaluation of coronary artery stenosis and the associated changes in strain values. Still, its reference value was reported only in a few studies and confined to the left chamber. Previously, Li et al. [15] reported a reference range of CT strain using the same software, and the result absolute LV global strain values were higher than those of ours, except for LV GCS (ranges for LV GLS: −26.6±3.2%; GCS: −22.7±3.0%; and GRS: 74.5±15.2%). However, the ranges were derived from the subjects without evidence of normal echocardiography. In another study of CT strain conducted with the same software, the normal LV GLS was −19.9% [24]. The reference ranges measured using feature tracking method with cardiac MR were LV GLS: 21.3±4.8%; GCS: −26.1±3.8%; and GRS: 39.8±8.3% [25] and by 3-dimensional speckle tracking echocardiography (3D STE) ranged as LV GLS: 15.80–23.40% (mean, −19.05%); GCS: 15.50–39.50% (mean, −22.42%); and GRS: 19.81–86.61% (mean, −47.48%) [26]. Because complete exclusion of papillary muscles and trabeculations from the endocardial border on short-axis imaging during the systolic phase is difficult, the range of LV GRS might be larger among studies. Despite the differences in modalities and subsequent differences in temporal resolution, our results are similar to those from previous CMR or echocardiography studies [25, 26].

In regional and segmental values, the LV longitudinal strain, transverse strain, and inward displacement were different according to the apical to basal segments and sides of walls: an increment in the apicobasal direction of LV longitudinal strain, highest value in middle segments of both LV transverse strain and inward displacement, and larger longitudinal strain values in lateral segments than those of medial segments. This heterogeneity of strain values has been noted in previous studies with 3D STE [27] and CT [15], reflecting the regional morphologic and functional difference of LV myocardium [28]. On the other hand, the inward displacement showed similar regional values and trends to transverse strain. This parameter could be a potential assistant parameter for segmental transverse strain, which offers a low interobserver reproducibility [29].

Only a few studies have examined CT strain for other chamber parameters. Szilveszter et al. [24] reported LA CT strain but in post-transcatheter aortic valve implantation patients, of which the LA strain could be affected during the reverse LA remodeling process [30]. Recently, Sun et al. [31] reported a reference value for LA strain measured by 2-dimensional (2D) STE in a multicenter cohort consisting of the same race as our study, which was higher than our results (LA reservoir strain, 35.9±10.6% vs. 28.6±8.5%; LA pump strain, 13.9±3.6% vs. 13.2±6.4%, and LA conduit strain, 21.9±9.3% vs. 15.5±8.6%). To assess the LA strain with echocardiography, adequate image acquisition covering the whole LA is required. Therefore, without dedicated echocardiography acquisition, retrospective analysis is often limited, and even in the prospective acquisition, the poor sonic window could hamper the evaluation of LA strain. This makes strain analysis with echocardiography more restricted than with cardiac CT.

In terms of the RV strain parameters, 2D STE-measured absolute values [32] were higher than our results (RV GLS, −25.8±3.0% vs. −22.0±5.7; RV free wall strain, −30.5±3.9% vs. −26.9±6.4%; and RV septal strain, −20.1±3.2% vs. −16.2±6.5%). This tendency was the same in RA GLS; The 3D STE-measured values [33] were slightly higher than our results (RA GLS, 37.2% vs. 27.9%). As observed similarly in another CT strain study, that might be related to modality difference [24], in which CT has lower temporal resolution than echocardiography. However, CT has the advantage of more stable delineation of endocardial borders of cardiac chambers with its higher spatial resolution. Awareness of the reference range of CT strain from four cardiac chambers could help assess early cardiac deformation and understand the inter-chamber relationships in various diseases [34].

The age and sex-dependencies of strain parameters have been noted in all cardiac chambers, measured by echocardiography [34, 35], CMR [25], and CT [15]. However, there were some differences in each specific parameter. LV GLS is known that higher in women and decreases with the age [15, 35] but not in other reports [26, 34]. In our study, LV GLS and GCS were higher in women and negatively correlated with the age only in men, consistent with previous studies with 2D STE [35] and CT [15]. The absolute RV strain parameters, which were significantly higher in women and negatively correlated with age, except RV free wall strain, were also in line with a previous study of a 2D STE-measured RV strain [32]. Declines in LA strains with aging were reported in large cohort studies [36, 37]. However, in our study, there was no distinct correlation between demographics and LA strain except in LA conduit strain with sex-difference and correlation with age in LA reservoir strain of women. Finally, in RA, the GLS was higher and RA GLS positively correlated with the age in women, consistent with previous echocardiography studies [33, 34]. Addition et al. [34] reported higher longitudinal strains in all four chambers of women by 2D STE, and the tendency was the same in our result except for LA strains.

Regarding interobserver reproducibility, the lowest agreement in LV GRS has been noted in previous studies with CT [15, 38] and CMR [25]. The RV septal strain showed the second lowest relative agreement, but the absolute difference in the RV strain measurement was considerably slight (0.1). Overall, the longitudinal strain values showed acceptable interobserver reproducibility. Therefore, it is considered that CT strain could provide a reproducible measurement of representative strain parameters from all four chambers.

There are several limitations to our study. First, because this is a retrospective observational study, we could compare the CT strain values with echocardiography only in 34 patients who had assessable images, and the correlation with echocardiography-derived strain was lower than those reported in previous studies (0.60–0.79 [11, 12]). Because echocardiographic strain analysis was available in small number of subjects and retrospectively performed with non-dedicated sonic window for strain analysis, the results was lower than we expected. In actual clinical practice, echocardiographic images for strain analysis especially for both atria did not be sufficiently obtained for subjects judged to have normal cardiac function. It is expected that the correlation between the two modalities will be improved if the echocardiography images have been precisely taken for strain analysis. Second, we conducted this study in a single tertiary referral hospital, with a single race of subjects, and single software for post-processing, therefore, there is a possibility that the generalizability is restricted. Moreover, the number of subjects in each subgroup was small to provide reference values based on age/sex distributions. In this study, we were able to obtain multiphase cardiac CT data during a specific period for setting the health screening CT protocol. However, conducting multiphase cardiac CT in clinically normal individuals without arrhythmia cannot be justified from a radiation exposure standpoint, making it difficult to perform such studies in larger cohorts. Therefore, even though the number of subjects is small, the importance of this study lies in the provided normal CT strain values, which can serve as a reference in future research using CT strain to study various cardiac diseases. Although this study has several limitations, we authors believe that it has a value to suggest the reference CT strain values of four cardiac chambers, in recent clinical situations which CT stain have gradually received attention with a few available software for CT strain analysis. Further validations with larger cohorts and comparison with other software and modalities are of value. Additionally, the recent integration of artificial intelligence in CMR strain measurement [39] suggests a similar potential for CT strain analysis. This approach could reduce interobserver variability and streamline the process, enhancing the practicality of CT strain in clinical use.

In conclusion, the measurement of CT strain using the feature tracking method could provide strains with reproducible results in all four cardiac chambers. The influence of age and sex on strains varied across the chambers but was in line with previous observations with echocardiographic data. This could be helpful information for detecting early cardiac deformity and inter-chamber relationships with the concomitant use of CT coronary angiography.

## Supporting information

S1 Text Measurement of echocardiographic strain. (DOCX)

S2 Text Cardiac CT acquisition. (DOCX)

S3 Text Measurement of CT-derived cardiac strain. (DOCX)

S1 Table Interobserver reproducibility of CT measurement and strain. (DOCX)

S1 Fig Difference in between image reconstructed with 10% of R-R interval and 5% of R-R interval. (DOCX)

S2 Fig Diagram for the measurement of LV inward displacement. (DOCX)

S3 Fig Left ventricular CT strain parameters according to the age and sex. (DOCX)

S4 Fig Difference and correlation in between CT and echocardiography. (DOCX)

## Abbreviations

2D: 2-dimensional
3D: 3-dimensional
AHA: American Heart Association
CMR: cardiac magnetic resonance imaging
ECG: electrocardiography
EF: ejection fraction
FAC: fractional area change
GCS: global circumferential strain
GLS: global longitudinal strain
GRS: global radial strain
LA: left atrial
LV: left ventricular
LVEF: left ventricular ejection fraction
RA: right atrial
RV: right ventricular
STE: speckle tracking echocardiography
