# Supplementary material for: Reference ranges of computed tomography-derived strains in four cardiac chambers
Source: PLoS One. 2024 Jun 6;19(6):e0303986. doi: 10.1371/journal.pone.0303986 (PMC11156317; doi:10.1371/journal.pone.0303986)
Supplement: S1 Text — (DOCX) [file pone.0303986.s001.docx]

**Supporting information**

**S1 Text.**

**Measurement of Echocardiographic Strain**

The strain values in our study were obtained from two-dimensional echocardiography images, which were digitally stored at a rate of 60 frames/sec in a cine-loop format. An experienced cardiologist measured the strains using offline software (2D Cardiac Performance Analysis; TomTec Imaging Systems). The left ventricular (LV) endocardial border was manually delineated at end-systole using a point-and-click method. Subsequently, the software automatically tracked this border. We performed the analyses using all three apical views (LV four-chamber, two-chamber, and three-chamber) and computed the LV global longitudinal strain (GLS) as the average of the global values from these three views.

For the left atrial (LA) strains, we used the apical four-chamber view. The LA reservoir strain was determined at left ventricular end-diastole, and the pump strain was measured following the onset of the p-wave in the electrocardiogram, coinciding with the sharp downslope in the strain trace. We calculated the LA conduit strain as the difference between the strain value at the onset of atrial contraction and the peak strain value. The LA fractional area change (FAC) was computed using the formula: (area at end-systole − area at end-diastole)/area at end-systole, in the apical four-chamber view.

For the right ventricular (RV) GLS, we manually traced the endocardial border in the apical four-chamber view, similar to the LV GLS process. RV wall was divided into six segments for this analysis: basal free wall, mid-free wall, apical free wall, basal septum, mid-septum, and apical septum. The RV GLS was defined as the average of these six segments. The right atrial (RA) FAC was calculated in the same manner as the LA FAC.
