# Supplementary material for: Reference ranges of computed tomography-derived strains in four cardiac chambers
Source: PLoS One. 2024 Jun 6;19(6):e0303986. doi: 10.1371/journal.pone.0303986 (PMC11156317; doi:10.1371/journal.pone.0303986)
Supplement: S2 Text — (DOCX) [file pone.0303986.s002.docx]

**Supporting information**

**S2 Text.**

**Cardiac CT Acquisition**

Electrocardiography (ECG)-gated cardiac CT was performed using a second-generation dual-source CT scanner (Somatom Definition Flash, Siemens, Erlangen, Germany). Unless subjects were contraindicated to beta blockers, an oral dose of bisoprolol (2.5 mg, Concor, Merck, Darmstadt, Germany) was administered 1-hour before the CT scan in case of the heart rate was more than 75 beats/min. Two puffs (2.5 mg) of isosorbide dinitrate (Isoket Spray; UCB Pharma, Monheim, Germany) were also administered to the participants’ oral cavity for optimal dilatation of the coronary artery. ECG-based tube current modulation (a pulsing dose window of 70%–80% of the R-R interval) was applied during the retrospective ECG-gated spinal scan. The contrast material was administered as a bolus dose of 60–70 mL with a rate of 4.0 mL/s, followed by a saline chaser. The imaging parameters comprised beam collimation, 128 × 0.6 mm^2^; gantry rotation time, 280s; tube voltage, 80–120 kV; and tube current–exposure time product, 185–380 mA with automated dose modulation (CARE dose 4D; Siemens). The mean tube current (mAs) and dose-length product were 293.1 ± 36.5 and 485.8 ± 168.6 mGy · cm, respectively. We used a standard cardiac filter of a smooth convolution kernel (B26f) and a slice of 3 mm without a slice gap to reconstruct the images. The sets were reconstructed at every 10% increment in R-R interval, and the multiphase cardiac CT data were transported to commercial software (Medis) for post-processing. In order to demonstrate the effect of the number of reconstruction phases on CT strain measurement, we have included the information with the comparison of the four chamber strains between 10 and 20 reconstruction phases using additional 20 patients who acquired multiphase CT data due to valvular heart disease and available 20 reconstruction phases. The strain results showed mean difference ranged from -0.9 to 2.6 (95% LOA, from [-0.4–0.3] to [-4.1–9.2] (S1 Fig). LV GLS demonstrated the smallest mean difference (-0.1) and narrowest limits of agreement (-0.4–0.3).
