# Supplementary material for: Reference ranges of computed tomography-derived strains in four cardiac chambers
Source: PLoS One. 2024 Jun 6;19(6):e0303986. doi: 10.1371/journal.pone.0303986 (PMC11156317; doi:10.1371/journal.pone.0303986)
Supplement: S3 Text — (DOCX) [file pone.0303986.s003.docx]

**Supporting information**

**S3 Text.**

**Measurement of CT-derived Cardiac Strain**

First, for the LV strain, 4-, 2-chamber long-axis, and short-axis reconstruction images from multiphase data were generated by post-processing (Medis, 3D view). Using the Qmass package (version 8.1), the automatically drawn endocardial and epicardial contours were manually corrected from the mitral annulus to the apex on both visually selected end-diastolic (when the maximum size of left ventricular [1] cavity) and end-systolic (when the minimum size of LV cavity) phases while excluding papillary muscles and trabeculation from the myocardium. Then, LV myocardial border throughout the whole cardiac cycle were automatically tracked and LV strain values were calculated (QStrain package, version 4.1). Following LV strain parameters were obtained: global longitudinal (GLS), circumferential (GCS), and radial (GRS) strains, segmental values of the longitudinal strain (according to the 17-segment model of the American Heart Association [1]), transverse strain (radial direction of strain measured from long-axis view [Figure 1]), and inward displacement of endocardium derived from the long-axis images, and segmental values for radial and circumferential strains derived from the short-axis images. Inward displacement is a value defined for each endocardial border point, representing the displacement vector component directed toward the “center of contraction.” Such a center is defined as a point on the LV axis whose position ranges from one-half and two-thirds of the base-apex distance from the basal to the apical regions, respectively (S2 Fig).

For RV measurement, the endocardial contour was manually drawn from the tricuspid annulus to the apex in a 4-chamber long-axis image similar to LV strain analysis. RV GLS, segmental (free wall and septum) strain, and fractional area change (FAC) were obtained.

For the LA strain, LA endocardial wall was manually drawn on both end-diastolic and end-systolic phases of a 2-chamber long-axis image. The LA appendage and pulmonary veins were excluded from the selection of the LA endocardial border. The following LA strain parameters were measured: LA reservoir strain as an average peak longitudinal strain value on a 2-chamber view, LA pump strain as a second peak point of the cardiac cycle-strain curve, and LA conduit strain as a difference between LA reservoir strain and LA pump strain. LA volume was measured at the end-systolic phase. LA FAC and ejection faction (EF) were also calculated.

Lastly, for RA strain, RA endocardial wall was drawn, excluding the RA appendage. RA GLS was measured during the RA reservoir phase. RA volume was measured at the end-systolic phase. RA FAC and EF were also calculated.

A trained radiology technician performed adjustments before measurement to confirm the endocardial and epicardial margins. Moreover, two board-certificated radiologists (BLINDED with 5 and 11 years of experience in cardiovascular radiology) re-checked margins before calculating the strain analysis and manually corrected if it was inadequate. For interobserver agreement, one board-certificated radiologist (BLINDED) independently measured cardiac strains in randomly selected 20 subjects.

**Reference**

1. Sengupta PP, Korinek J, Belohlavek M, Narula J, Vannan MA, Jahangir A, et al. Left ventricular structure and function: basic science for cardiac imaging. J Am Coll Cardiol 2006;48:1988-2001
