# Supplementary material for: Reference ranges of computed tomography-derived strains in four cardiac chambers
Source: PLoS One. 2024 Jun 6;19(6):e0303986. doi: 10.1371/journal.pone.0303986 (PMC11156317; doi:10.1371/journal.pone.0303986)
Supplement: S1 Table — (DOCX) [file pone.0303986.s004.docx]

**Supporting informations**

**S1 Table. Interobserver Reproducibility of CT Measurement and Strain**

|  | Reader1 | Reader2 | Difference | *P* | 95% LOA | Relative differences (%) |
| --- | --- | --- | --- | --- | --- | --- |
| **Left ventricle** |  |  |  |  |  |  |
| Global longitudinal strain, % | −17.3 ± 3.1 | −17.5 ± 2.8 | 0.2 | 0.280 | −1.2, 1.5 | −1.3 |
| Global circumferential strain, % | −25.1 ± 4.3 | −26.4 ± 4.3 | 1.3 | 0.013 | −2.8, 5.4 | −5.7 |
| Global radial strain, % | 45.5 ± 13.0 | 59.7 ± 13.2 | −14.3 | 0.000 | −39.5, 10.9 | −38.0 |
| EF, % | 51.0 ± 6.7 | 52.8 ± 6.1 | −1.8 | 0.005 | −6.9, 3.3 | −4.0 |
| **Right ventricle** |  |  |  |  |  |  |
| Global longitudinal strain, % | −19.2 ± 7.1 | −18.9 ± 6.2 | −0.3 | 0.487 | −4.4, 3.7 | −0.5 |
| RV free wall strain, % | −24.4 ± 8.6 | −23.7 ± 7.9 | −0.7 | 0.180 | −5.4, 3.9 | 2.1 |
| RV septal strain, % | −13.8 ± 7.4 | −13.7 ± 6.3 | −0.1 | 0.929 | −5.9, 5.8 | −12.7 |
| FAC, % | 33.2 ± 10.9 | 32.5 ± 10.2 | 0.6 | 0.343 | −5.1, 6.4 | 1.1 |
| **Left atrium** |  |  |  |  |  |  |
| LA reservoir strain, % | 24.6 ± 7.2 | 23.7 ± 6.3 | 1.0 | 0.086 | −3.7, 5.6 | 1.9 |
| LA pump strain, % | 11.5 ± 7.1 | 11.1 ± 7.0 | 0.4 | 0.050 | −1.4, 2.2 | 3.6 |
| LA conduit strain, % | 13.1 ± 6.8 | 12.6 ± 6.3 | 0.5 | 0.310 | −3.9, 5.0 | 0.5 |
| LA volume, mL | 67.2 ± 11.8 | 64.4 ± 11.3 | 2.8 | <.001 | −2.0, 7.7 | 4.1 |
| FAC, % | 32.7 ± 6.2 | 32.7 ± 5.6 | 0.0 | 0.985 | −4.7, 4.7 | −0.8 |
| EF, % | 44.9 ± 7.3 | 44.9 ± 7.3 | 0.1 | 0.913 | −5.7, 5.8 | 0.0 |
| **Right atrium** |  |  |  |  |  |  |
| Global longitudinal strain, % | 25.2 ± 11.9 | 23.5 ± 11.3 | 1.7 | 0.046 | −5.1, 8.5 | 5.7 |
| RA volume, mL | 66.1 ± 32.6 | 65.0 ± 31.3 | 1.1 | 0.119 | −4.7, 6.9 | 0.9 |
| FAC, % | 31.6 ± 10.9 | 30.4 ± 10.9 | 1.2 | 0.044 | −3.7, 6.0 | 3.7 |
| EF, % | 39.6 ± 14.3 | 37.9 ± 13.7 | 1.7 | 0.036 | −4.9, 8.2 | 4.0 |

EF = ejection fraction, FAC = fraction area change, LA = left atrium, LOA = limits of agreement, LV = left ventricle, RA = right atrium, RV = right ventricle
