# Supplementary material for: Reference ranges of computed tomography-derived strains in four cardiac chambers
Source: PLoS One. 2024 Jun 6;19(6):e0303986. doi: 10.1371/journal.pone.0303986 (PMC11156317; doi:10.1371/journal.pone.0303986)

**Supporting information**

**S1 Fig. Difference in between image reconstructed with 10% of R-R interval and 5% of R-R interval**

Bland-Altman plots for strain result differences of (A) LV GLS, (B) LV GCS, (C) LV GRS, (D) LA reservoir strain, (E) RA GLS, and (F) RV GLS measured from images reconstructed 10% of R-R interval and 5% of R-R interval. GCS = global circumferential strain, GLS = global longitudinal strain, GRS = global radial strain, LA = left atrium, LV = left ventricle, RA = right atrium, RV = right ventricle, SD = standard deviation.

(A) (B)


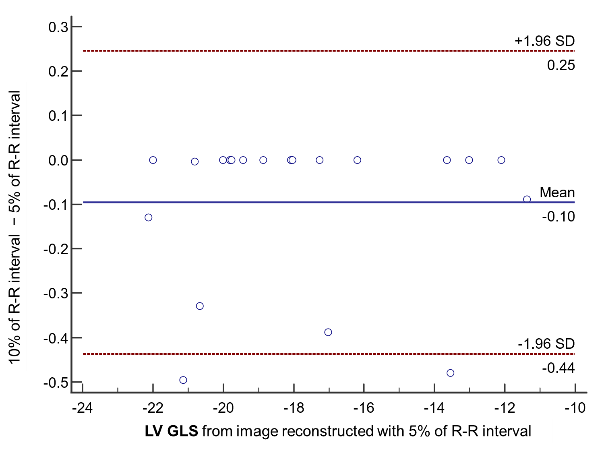

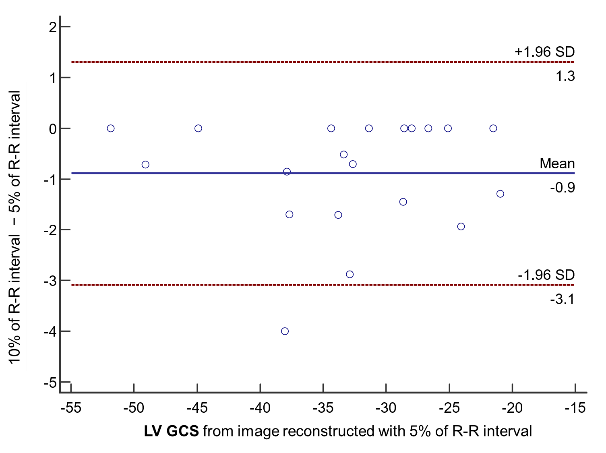


(C) (D)


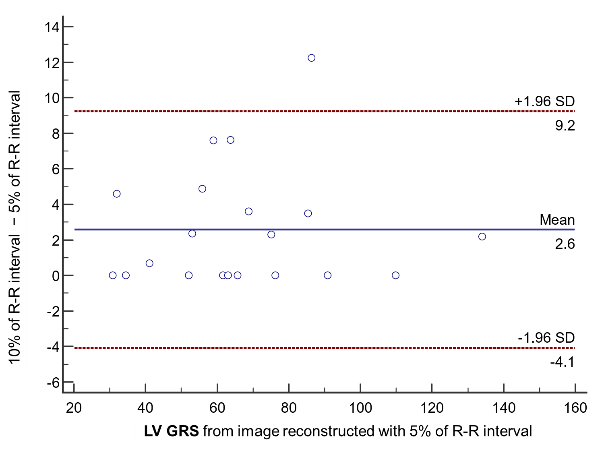

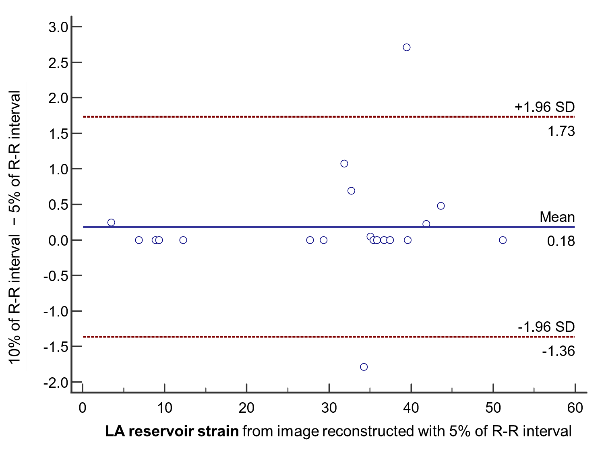


(E) (F)


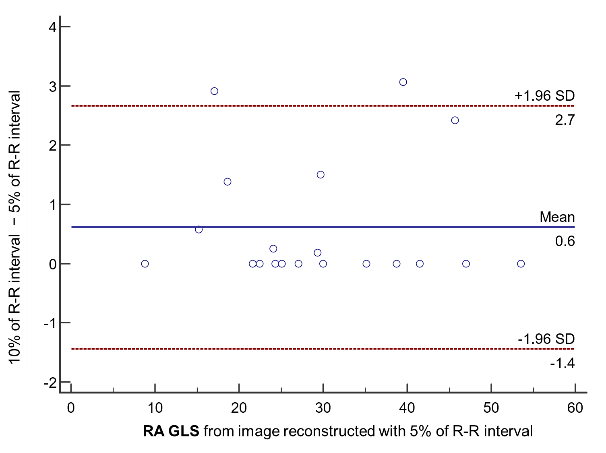

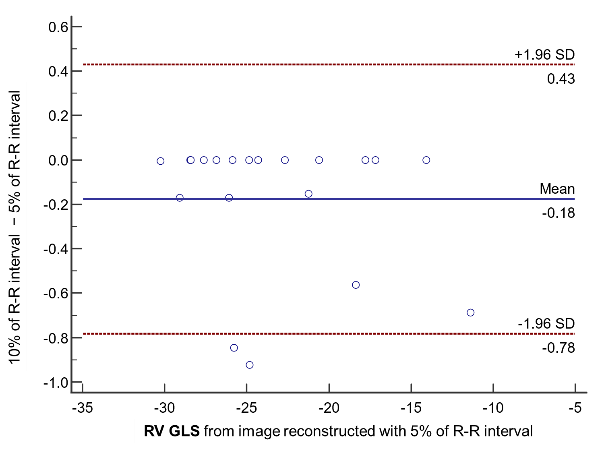

Supplement: S1 Fig — (DOCX) [file pone.0303986.s005.docx]
