# Supplementary material for: Reference ranges of computed tomography-derived strains in four cardiac chambers
Source: PLoS One. 2024 Jun 6;19(6):e0303986. doi: 10.1371/journal.pone.0303986 (PMC11156317; doi:10.1371/journal.pone.0303986)

**Supporting information**

**S2 Fig. Diagram for the measurement of left ventricular (LV) inward displacement.** The LV inward displacement represents the actual inward motion of each point toward the LV centerline (red bar), where ranges from one-half and two-thirds of the base-apex distance from the basal to the apical regions, respectively. The value is measured starting from the end-diastolic frame, assumed as the rest position, and increases during systole to reach a positive peak value at end systole. The distance to the LV center is expressed in percentage, where 0% means no contraction and 100% corresponds to a theoretical limit of a regional end-systolic size that shrinks to zero.


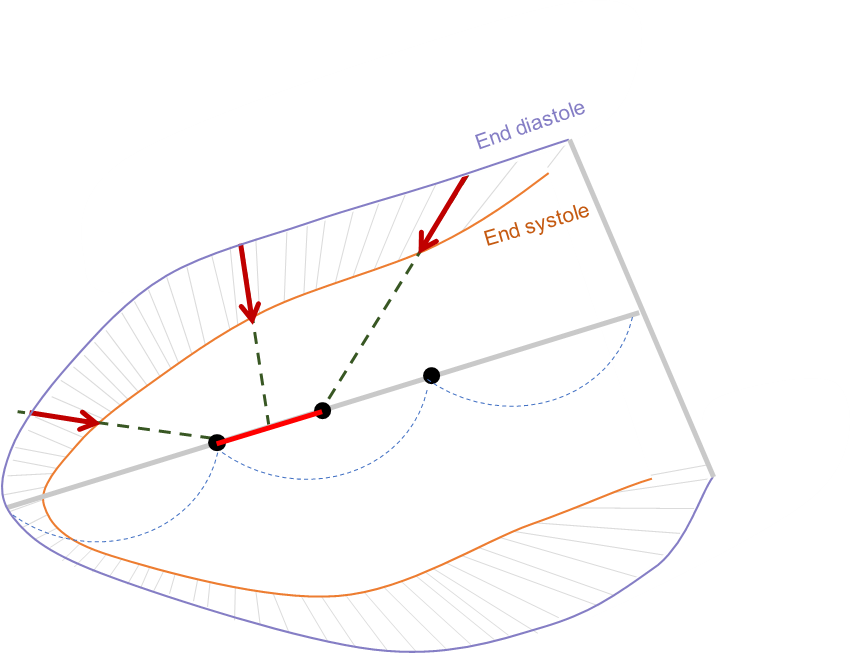

Supplement: S2 Fig — (DOCX) [file pone.0303986.s006.docx]
