# Supplementary material for: Reference ranges of computed tomography-derived strains in four cardiac chambers
Source: PLoS One. 2024 Jun 6;19(6):e0303986. doi: 10.1371/journal.pone.0303986 (PMC11156317; doi:10.1371/journal.pone.0303986)

**Supporting information**

**S3 Fig. Left ventricular CT strain parameters according to the age and sex.** The bar graph shows an average of absolute values of left ventricular global longitudinal, circumferential, and radial strain according to age group and sex. GCS = global circumferential strain, GLS = global longitudinal strain, GRS = global radial strain.


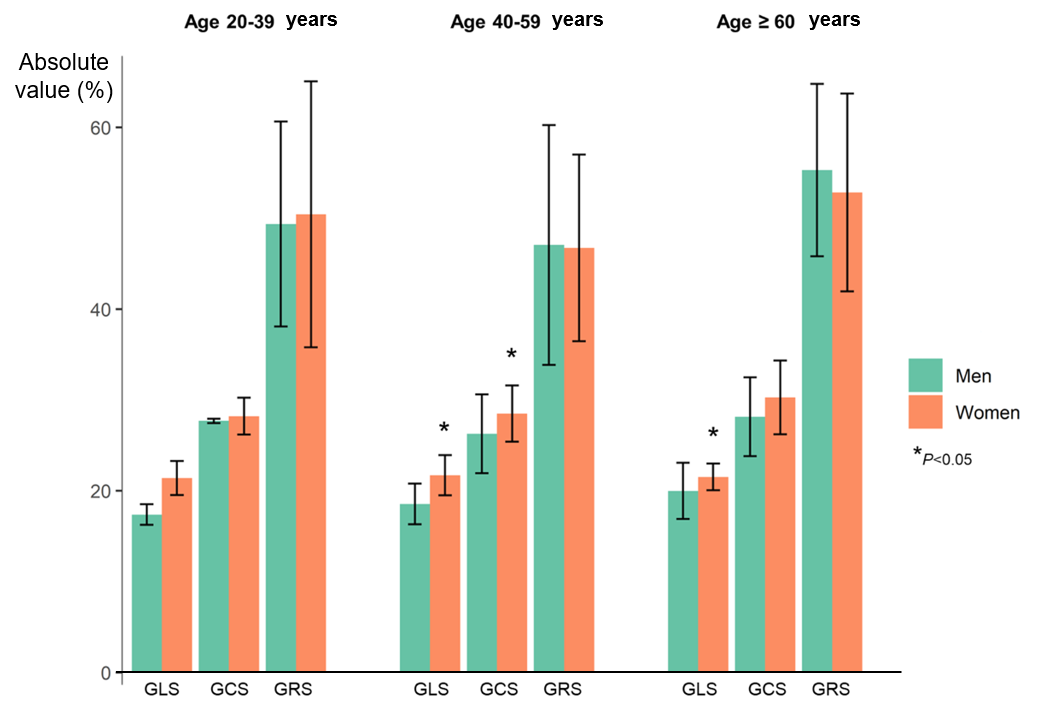

Supplement: S3 Fig — (DOCX) [file pone.0303986.s007.docx]
