# Supplementary material for: Reference ranges of computed tomography-derived strains in four cardiac chambers
Source: PLoS One. 2024 Jun 6;19(6):e0303986. doi: 10.1371/journal.pone.0303986 (PMC11156317; doi:10.1371/journal.pone.0303986)
Supplement: S4 Fig — (DOCX) [file pone.0303986.s008.docx]

**Supporting information**

**S4 Fig. Difference and correlation in between CT and echocardiography.** Bland-Altman plots and linear regression graphs for strain result differences of (A) LV GLS, (B) LA reservoir strain, (C) LA pump strain, (D) LA conduit strain, (E) LA FAC, (F) RV GLS, and (G) RA FAC measured from CT and echocardiography. FAC = fraction area change, GLS = global longitudinal strain, LA = left atrium, LV = left ventricle, RV = right ventricle, SD = standard deviation.

(A)


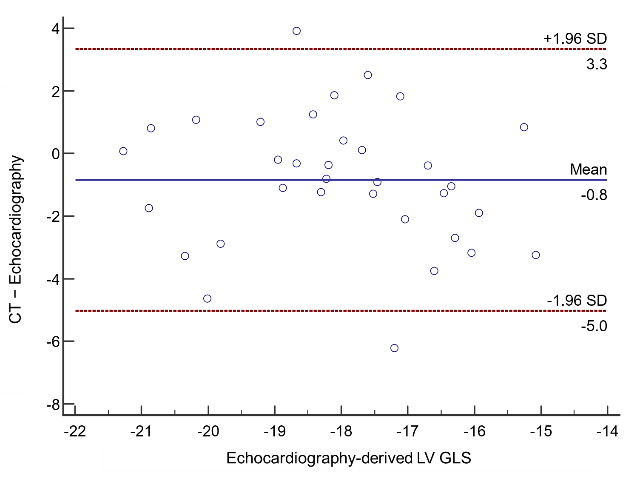

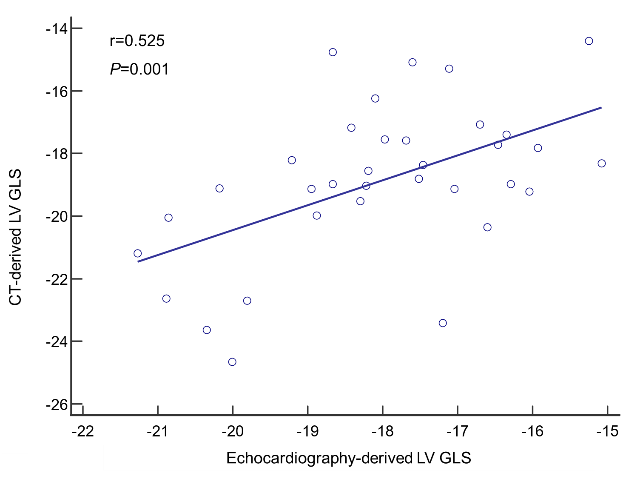


(B)


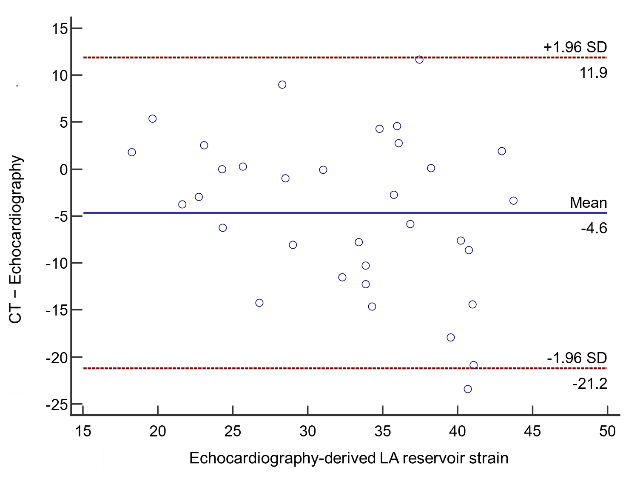

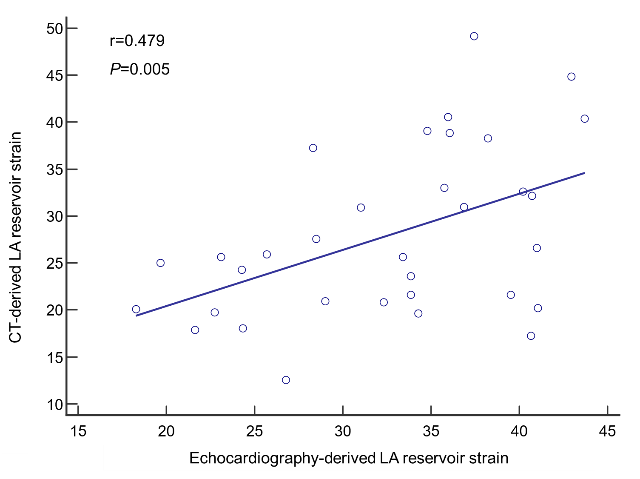


(C)


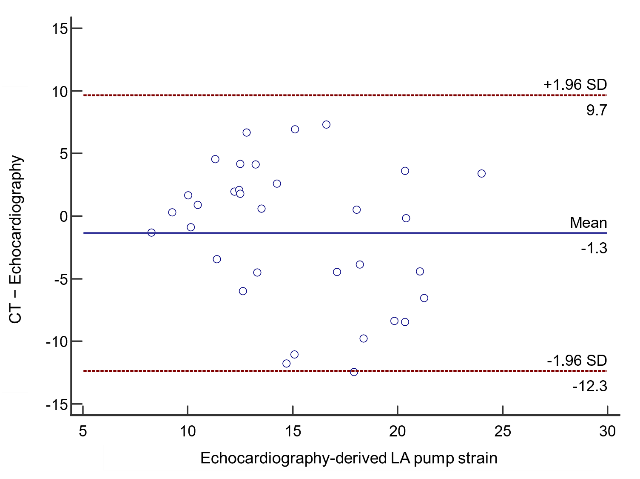

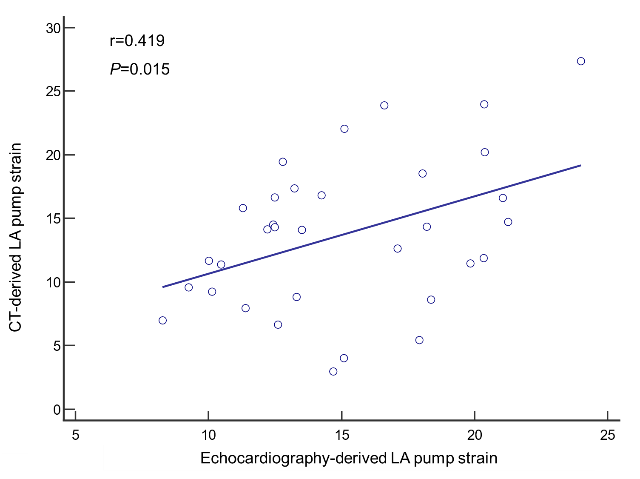


(D)


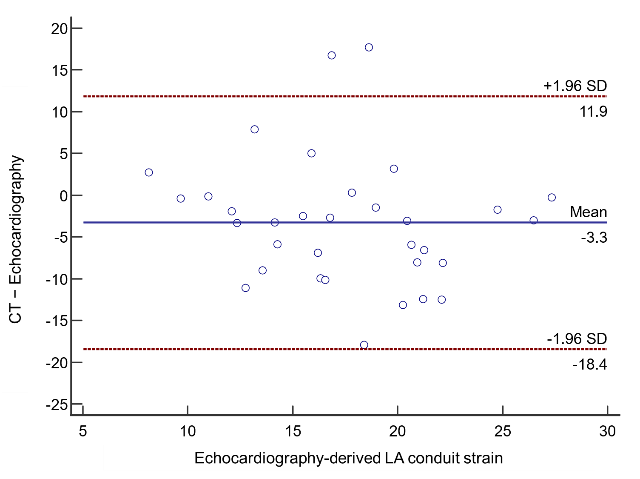

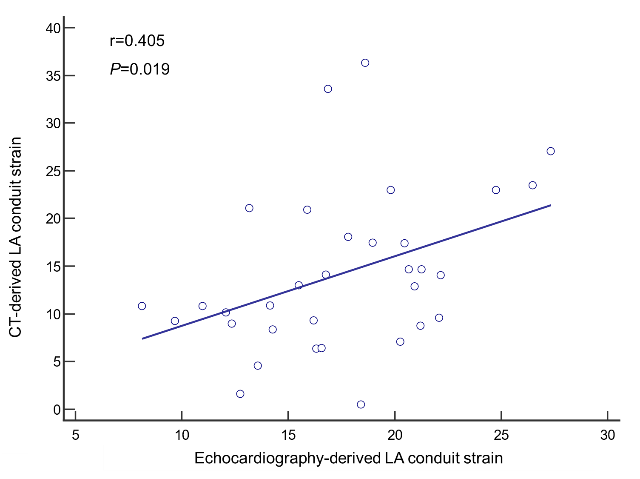


(E)


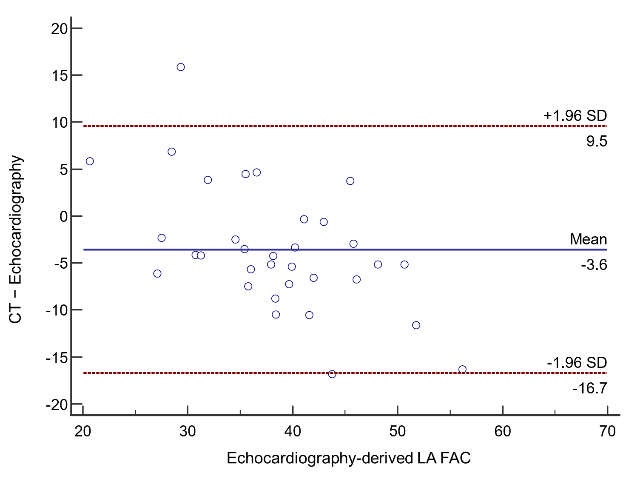

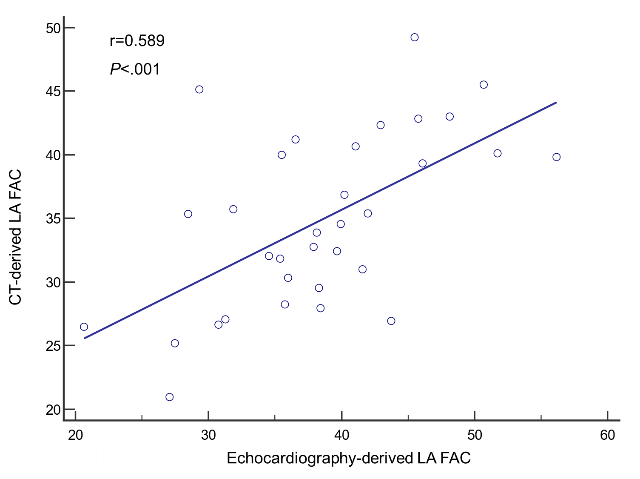


(F)


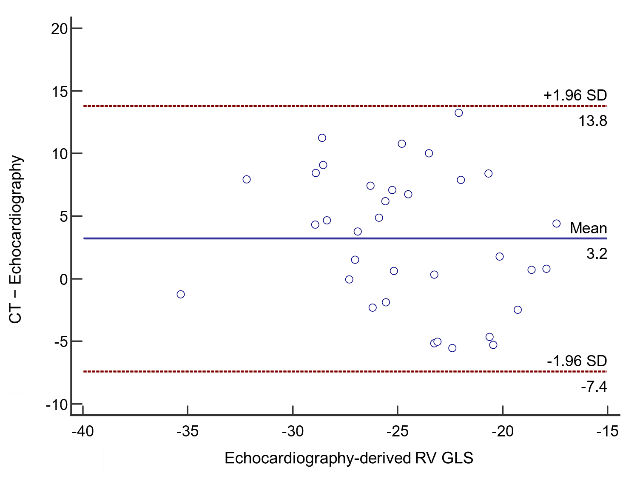

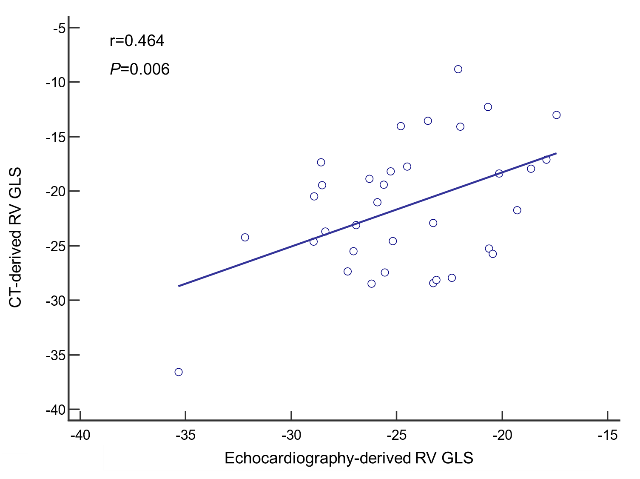


(G)


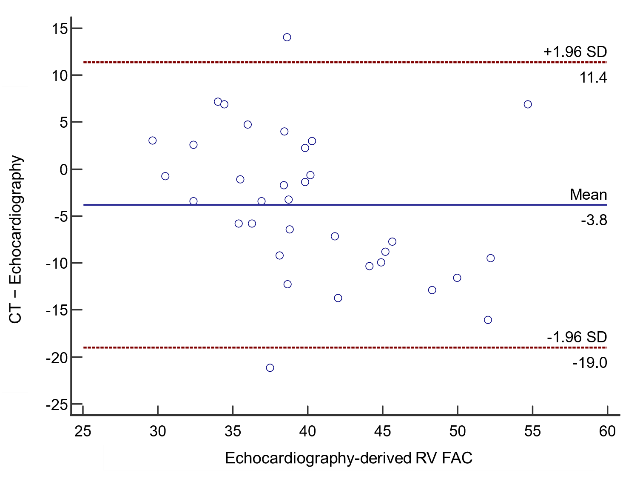

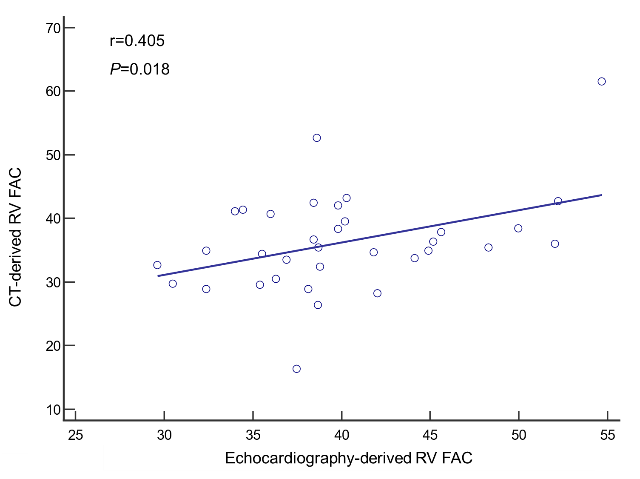


1. Szilveszter B, Nagy AI, Vattay B, Apor A, Kolossváry M, Bartykowszki A, et al. Left ventricular and atrial strain imaging with cardiac computed tomography: Validation against echocardiography. *J Cardiovasc Comput Tomogr* 2020;14:363-369
